# The impact of COVID-19 on the motivations of women seeking a uterus transplant

**DOI:** 10.2144/fsoa-2022-0047

**Published:** 2023-03-28

**Authors:** Saaliha Vali, Benjamin P Jones, Srdjan Saso, J Richard Smith

**Affiliations:** 1 Department of Surgery & Cancer, Hammersmith Hospital, Imperial College London, Du Cane Road, London, W12 0NN, UK; 2 Cutrale Perioperative & Ageing Research Group, Imperial College London, London, UK; 3 West London Gynaecological Cancer Centre, Hammersmith Hospital, Imperial College NHS Trust, London, W12 0HS, UK

**Keywords:** COVID-19, infertility, MRKH, pregnancy, Uterus transplant

## Abstract

**Aim:**

The aim of this study was to investigate the change if any, in the motivations of women seeking a UTx and determine the impact of the COVID-19 pandemic.

**Methods:**

A cross-sectional survey.

**Results:**

59% of women answered they were more motivated in achieving a pregnancy following the COVID-19 pandemic. 80% strongly agreed or agreed the pandemic had no impact on their motivation for a UTx, and 75% strongly agreed or agreed their desire for a baby strongly outweighs the risks of undergoing a UTx during a pandemic.

**Conclusion:**

Women continue to express a high level of motivation and desire for a UTx despite the risks imposed by the COVID-19 pandemic.

## Tweetable abstract

For women seeking a uterus transplant, the pandemic had little impact on their motivation desire for a transplant despite the risks imposed by the COVID-19 pandemic.

Over 200 women contacted the uterus transplant (UTx) UK team seeking a UTx between 2012 and 2019. The UK clinical trial named the Investigational Study into Uterine Transplantation (INSITU) trial received ethical approval in 2019 for ten women to undergo deceased donor uterus transplantation. This will involve retrieval of the uterus during a multi-organ retrieval from a brainstem dead donor. The feasibility of this procedure has been proven with 18 cases and five livebirths published [1]. The INSITU trial was in active recruitment phase and was live with the UK national organ donation network prior to the COVID-19 pandemic. In March 2020 the decision was made to suspend the study in light of the imposed, largely unknown risks in potential UTx recipients, particularly in light of the necessity to take immunosuppression. There were a number of women mid-way through their pre-operative investigations, the results of which would later be used to determine their eligibility into the trial. This uncertain period of waiting is likely to have resulted in significant stress among participants. Additionally, the cessation of undertaking fertility treatments also had an impact on participants given a prerequisite for entry onto the transplant programme was successful cryopreservation of five high grade embryos.

Infertility is widely known to result in significant psychological distress, often provoking feelings of anger, depression and anxiety [2,3]. These reactions may be intensified during a pandemic, and may result in significant feelings of despair [4].

The main cohort seeking a UTx are women with absolute uterus factor infertility (AUFI). This group includes women with a congenital absence of the uterus termed Mayer–Rokitansky–Küster–Hauser Syndrome (MRKH), women who have undergone a hysterectomy and women with a uterus which is incapable of carrying a pregnancy such as the case with severe Ashermans syndrome. The majority of UTx recipients to date have been women with MRKH [5]. Patients with MRKH often present in puberty around the age of 16 years with amenorrhoea in the presence of normal secondary pubertal characteristics. This diagnosis often impacts on their sense of wellbeing and quality of life [6]. The discovery of the loss of childbearing at this early stage of their life has a profound impact on their psychological wellbeing and remains so as they progress through into adulthood [7,8]. Likewise, for the remaining AUFI cohort, the diagnosis also results in immediate long-lasting negative perceptions mostly relating to the loss of gestation [9].

The alternative options for women with AUFI to acquire motherhood included surrogacy and adoption. The emergence of UTx has enabled them to embrace the hope of biological, gestational and legal motherhood. A global pandemic resulting in the halting of non-essential surgery is therefore likely to have and had negative consequences on the hope carried by this cohort.

Understandably, the impact of COVID-19 on recipients of solid organ transplants (SOT) is more significant than that of the general population. Mortality rates in transplant recipients infected with COVID-19 have been shown to be higher than the general population [10–12]. In one large scale study of 9845 kidney transplant recipients, 66% of the hospitalised patients were male with a mean age of 60 years. Comorbidities were highly prevalent with 95% suffering with hypertension, 52% with diabetes and 49% with obesity [13]. This demographic is largely disparate from the UTx cohort who are young and healthy women, and by virtue of the selection criteria, without significant medical commodity. Thus, the risk profile for UTx candidates is likely to be very different to the general SOT population.

## Main objective of study

Being a quality of life improving transplant procedure, and owing to the uncertainties surrounding risk, the pause on UTx activity during the COVID-19 pandemic was appropriate. However, the often overlooked potential psychological implications on such women also required consideration. The once given ‘hope’ of parenthood for women given a diagnosis of AUFI was, during the pandemic, repressed. To investigate the impact upon those women considering undergoing UTx, we devised an electronic questionnaire-based study. The aim of this study was to investigate the change if any, in the motivations of women seeking a UTx and determine the impact of the COVID-19 pandemic.

## Method

### Eligibility

The study invitation was distributed electronically via email. Women who had previously contacted the Womb Transplant UK team seeking information or who submitted an application for the womb transplant trial were sent an invitation. The inclusion criteria was women over the age of 16 years who were seeking or who were interested in a UTx for themselves. Additionally, only women fluent in English were included. The only exclusion criteria was women who did not meet the minimum age criteria. An email was sent to all women on our records meeting the inclusion criteria containing a short brief on the study and with an attached patient information leaflet (Supplementary Materials 1). The email contained a link to the electronic consent form which required completion prior to proceeding to the electronic questionnaire. Participants were recruited over a four-month period between April 2021 and July 2021.

### Study design

The questionnaire was constructed on the SurveyMonkey^®^ platform and consisted of 45 items recording demographic information followed by questions on the impact of COVID-19 on: the desire for a UTx, the choice of uterus donor type, fertility treatment and the views of the partner (Supplementary Materials 2). All questions were closed, with the option to add comments if warranted. A five-point Likert scale was employed for questions relating to perceptions and beliefs. There was no personally identifiable data collected and thus all responses remained anonymous.

### Outcome measures

The impact of COVID-19 on the motivations of women seeking a womb transplant.

### Statistical analysis

Descriptive data analysis was performed. Quantification of dataset was performed through percentages and calculation of the mean and the mode. Quantification of the responses achieved on the Likert scale was performed by assigning a numerical score ranging from 0 to 5 to each of the five items presented. A score of 5 was assigned to ‘strongly agree’, and a score of 1 was assigned to strongly disagree in a graduated manner.

## Results

A total of 171 women were contacted to take part. A total of 102 women consented to take part in the study and a total of 96 women subsequently completed the questionnaire.

The overall response rate was 56%. The majority of respondents were aged between 30–39 years (n = 54, 56%), were of white ethnicity (n = 81, 84%) and of the Christian faith (n = 41 43%). Eighty- seven women (91%) specified they were married or living with a partner. Characteristics of participants are shown in Table 1.

**Table 1.** Demographic details of participants.

Age, range (years)	n	%
16–19	0	0%
20–29	31	32%
30–39	54	56%
40–49	9	9%
50–59	2	2%
Total	96	
**Ethnicity**		
White	81	84%
Asian	10	10%
Black	2	2%
Mixed	0	0%
Other	2	2%
Chosen not to answer	1	1%
Total	96	
**Employment status**		
Employed (full time)	63	66%
Employed (part time)	9	9%
Self Employed	11	11%
Housewife	3	3%
Unemployed	3	3%
Student	3	3%
Would rather not say	4	4%
Total	96	
**Educational attainment**		
No formal qualification	12	13%
Level 1 (1–4 GCSEs, Scottish Standard Grade or equivalent qualifications)	6	6%
Level 2 (5 + GCSEs, Scottish Higher, Scottish Advanced Higher or equivalent qualifications)	10	10%
Apprenticeship	2	2%
Level 3 (2 + A-levels, HNC, HND, SVQ level 4 or equivalent qualifications)	19	20%
Level 4 or above (first or higher degree, professional qualifications or other equivalent higher education qualifications)	36	38%
Other qualifications	11	11%
Total	96	
**Religion**		
Christian	41	43%
Muslim	9	9%
Hindu	1	1%
Other	13	14%
Atheist	16	17%
Would rather not say	16	17%
Total	96	
**Relationship status**		
Single	5	5%
Living with partner	46	48%
Married	41	43%
Divorced	0	0%
Separated	1	1%
Widowed	1	1%
Would rather not say	2	2%
Total	96	

In two thirds of women the cause of AUFI was MRKH (n = 64, 67%], while 28 women (29%) had undergone a hysterectomy. Twenty-three women (24%) had pre-existing children and of these, 52% (n = 11) had one child. In this group who had children, the modal route toward motherhood was having given birth to their child, while 13% (n = 3) reported they had their child via a surrogate.

**Figure 1.**
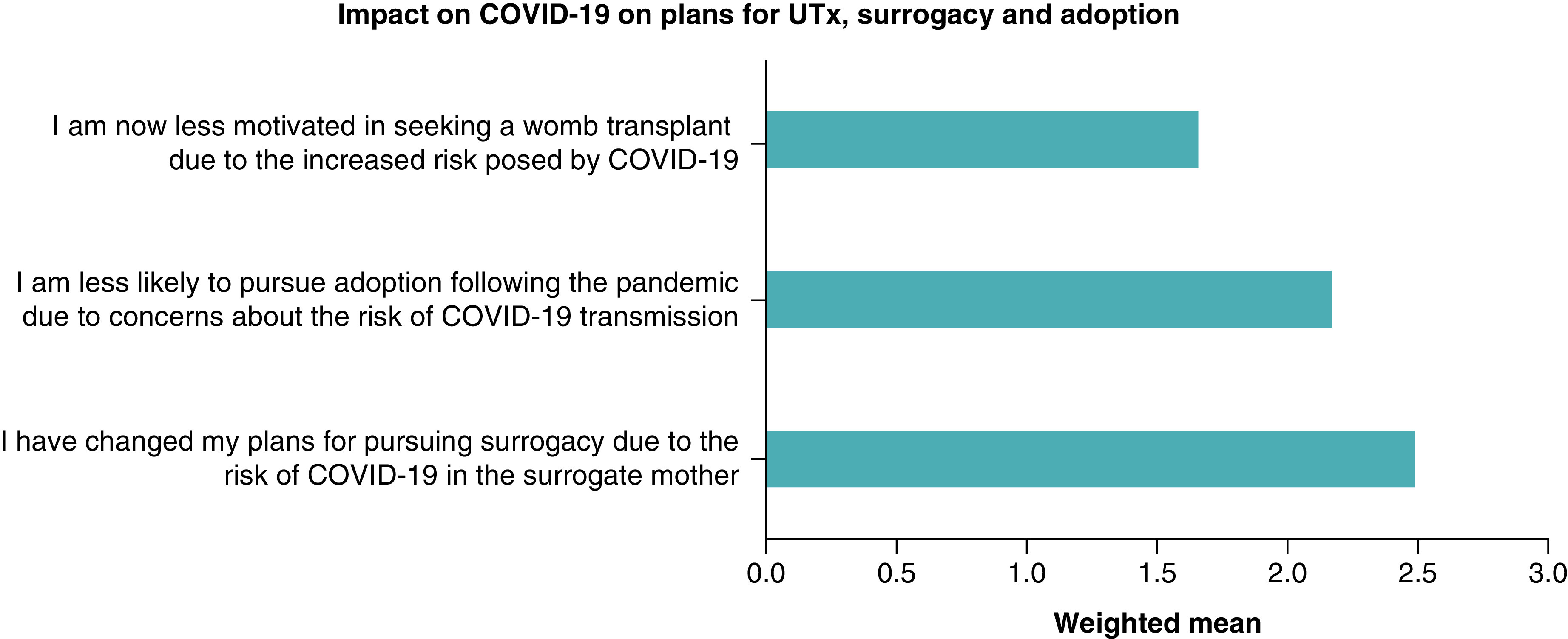
Impact on COVID-19 on plans for uterus transplant, surrogacy and adoption. UTx: Uterus transplant.

When asked about the route they were pursuing for parenthood, 83 women (86%) were actively pursuing a UTx, while 45% (n = 43) and 22% (n = 21) were pursuing surrogacy and adoption respectively. Of the women planning or actively pursuing surrogacy, 85% reported they had not changed their plans due to the risk of COVID-19 in the surrogate mother. With regards to adoption, women were asked if they were more likely to pursue this option due to their concerns on the risk of COVID-19 transmission with surrogacy or a UTx and 69% (n = 65) disagreed or strongly disagreed. Figure 1 displays the weighted mean of each response in relation to delaying the path toward parenthood during the pandemic.

**Figure 2.**
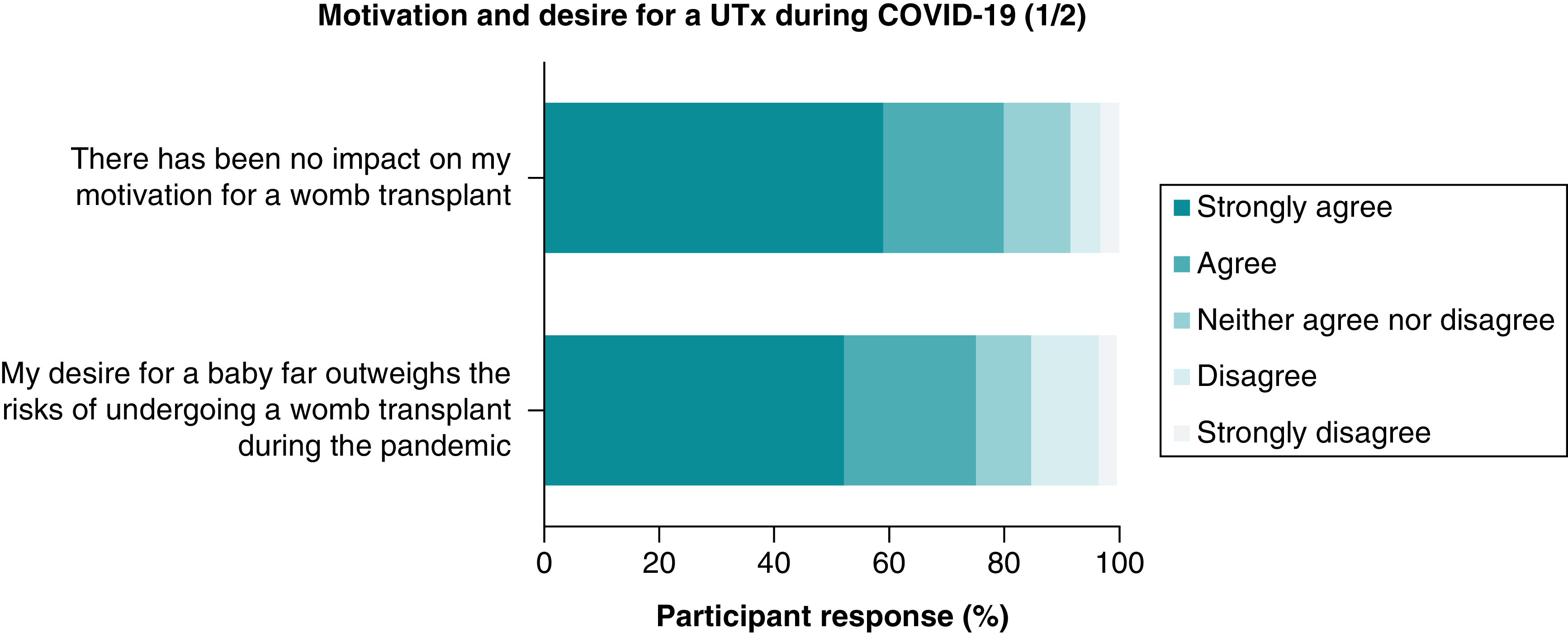
Motivation and desire for a uterus transplant during the COVID-19 pandemic (1/2). UTx: Uterus transplant.

**Figure 3.**
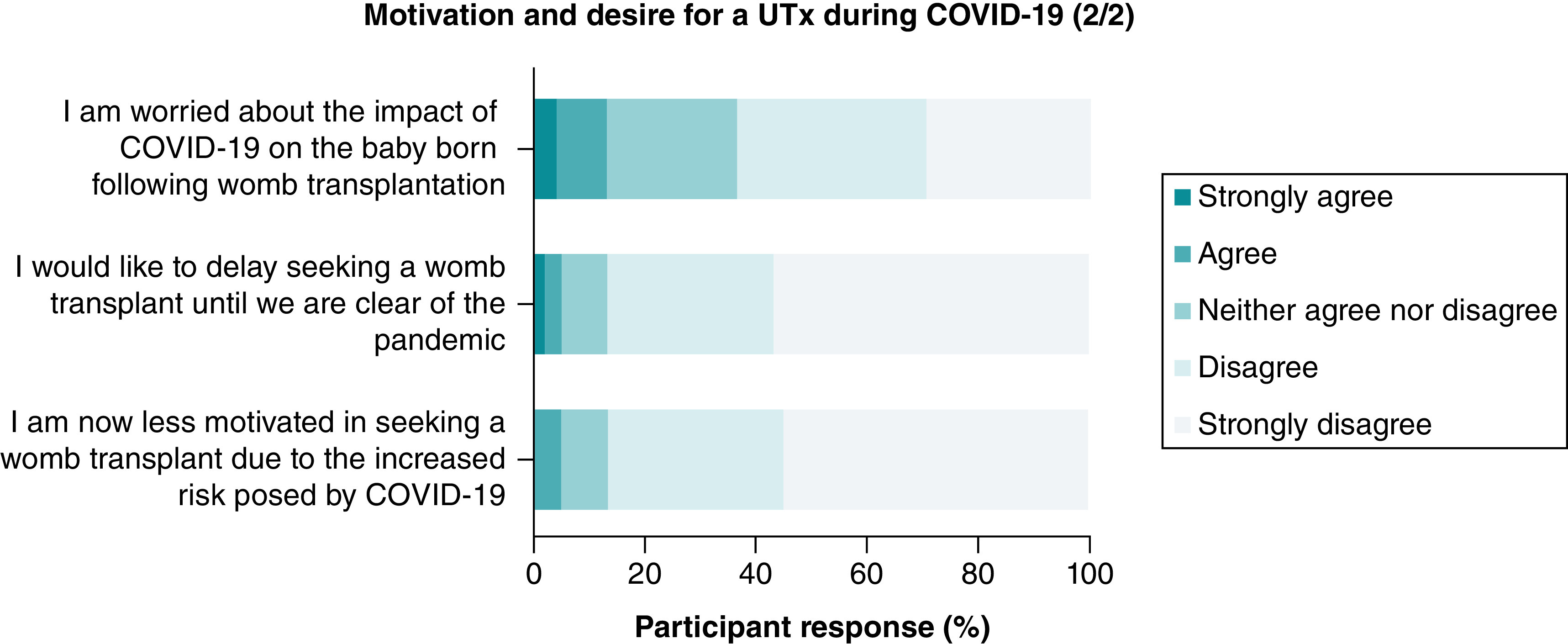
Motivation and desire for a uterus transplant during the COVID-19 pandemic (2/2). UTx: Uterus transplant.

Over half (n = 55, 59%) of the women answered they were more motivated in achieving a pregnancy following the COVID-19 pandemic and only 5% (n = 5) stated they were less motivated due to the increased risks COVID-19 posed in pregnancy (Figure 2). With regards to the impact on their motivation for a UTx, 80% (n = 76) strongly agreed or agreed the pandemic had no impact and 75% (n = 71) strongly agreed or agreed their desire for a baby strongly outweighs the risks of undergoing a UTx during a pandemic (Figure 3). 81% (n = 69) of women strongly disagreed or disagreed that their partner felt they should not pursue a UTx due to the risks imposed by COVID-19.

**Figure 4.**
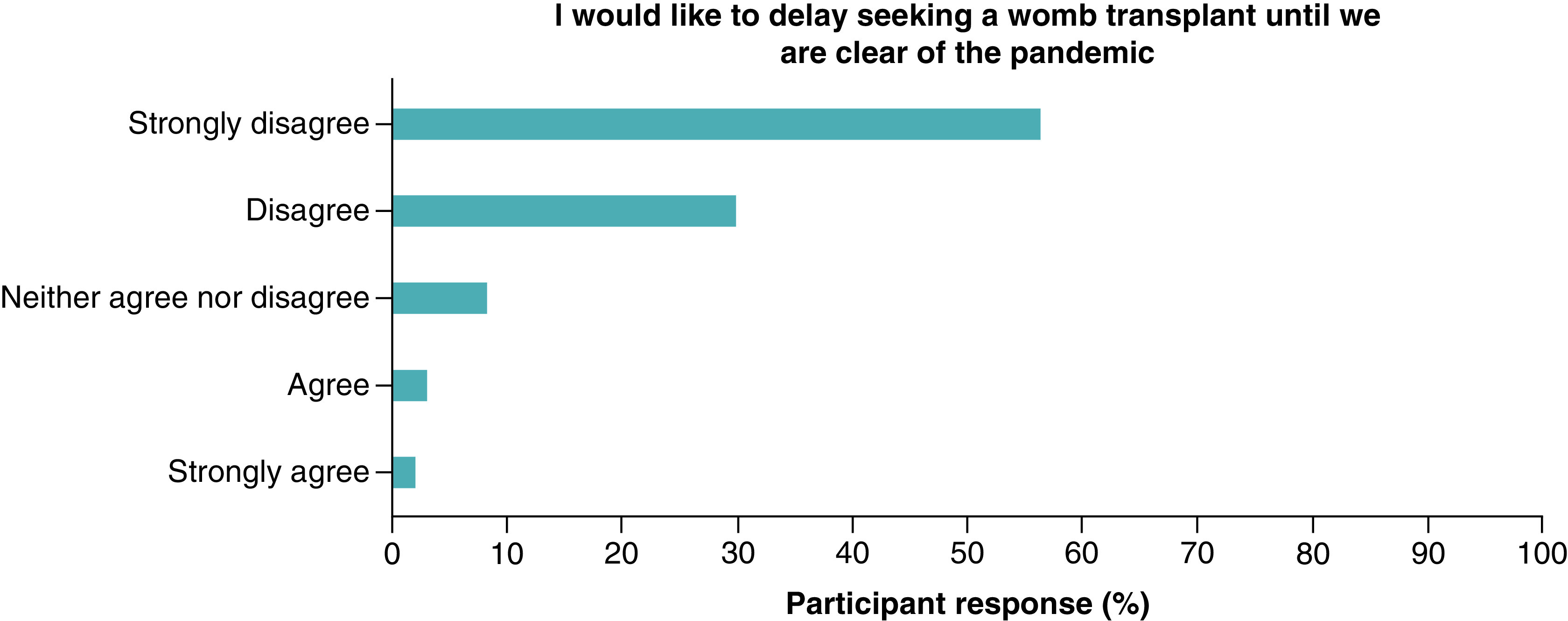
Women's views on delaying to pursue a uterus transplant until after the COVID-19 pandemic.

**Figure 5.**
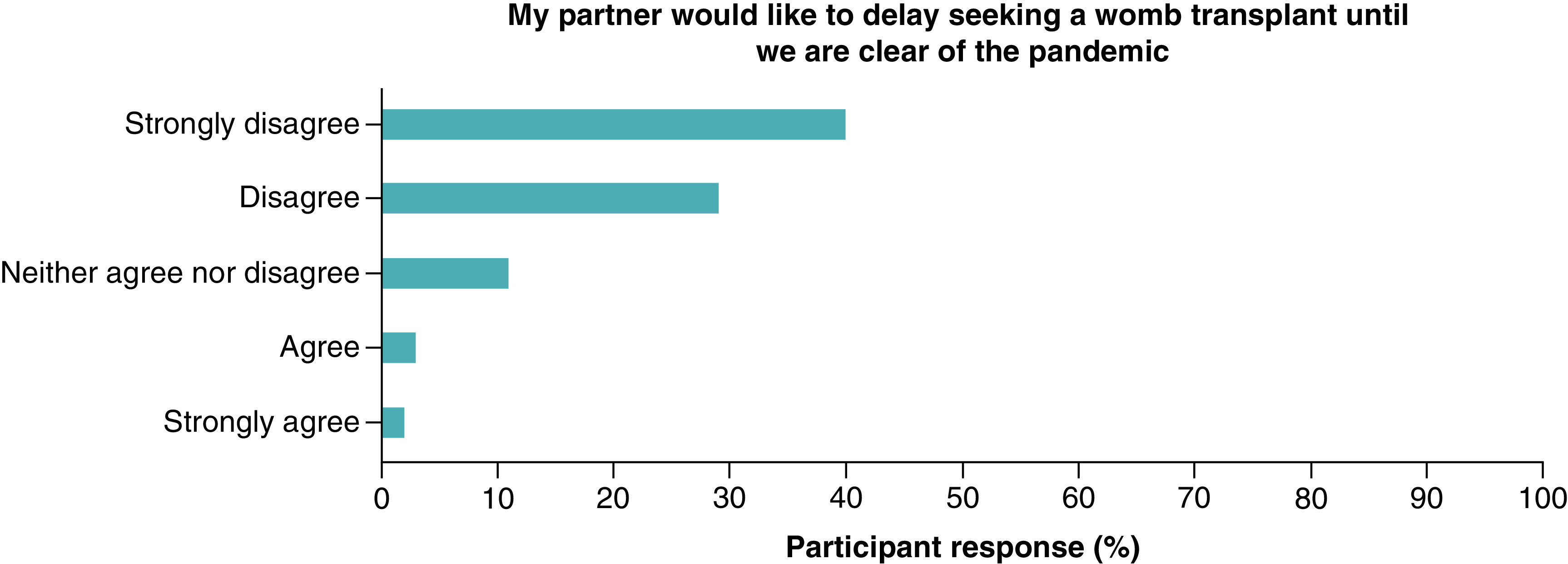
Partner's views on delaying to pursue a uterus transplant until after the COVID-19 pandemic.

**Figure 6.**
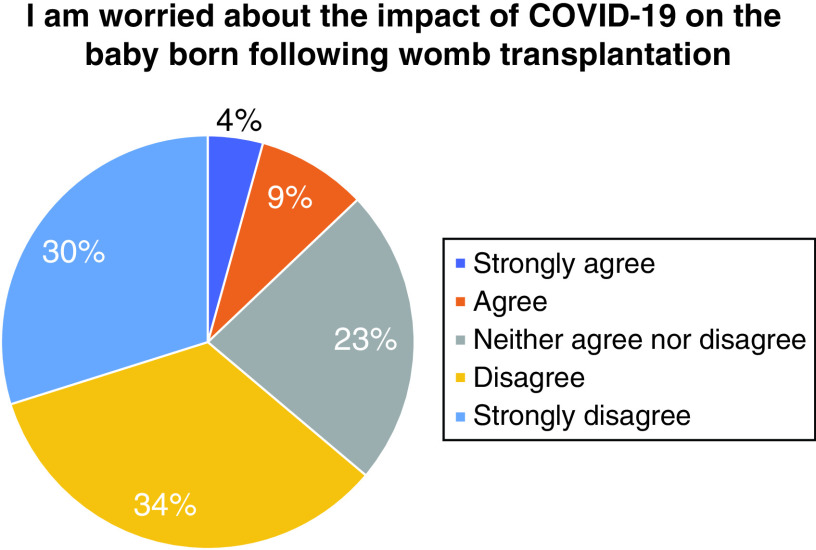
Concern on the impact of COVID-19 on the baby born following uterus transplantation.

Most of the women (n = 84, 87%) did not wish to delay seeking a UTx until the pandemic is over, with only 5% (n = 5) stating they strongly agreed or agreed to a delay (Figure 4). Partners views also reflected this with 81% selecting they strongly disagree or disagree with a delay (Figure 5). Almost two thirds of women (n = 60, 64%) strongly disagreed or disagreed on being worried about the impact of COVID-19 on the baby born following UTx (Figure 6).

**Figure 7.**
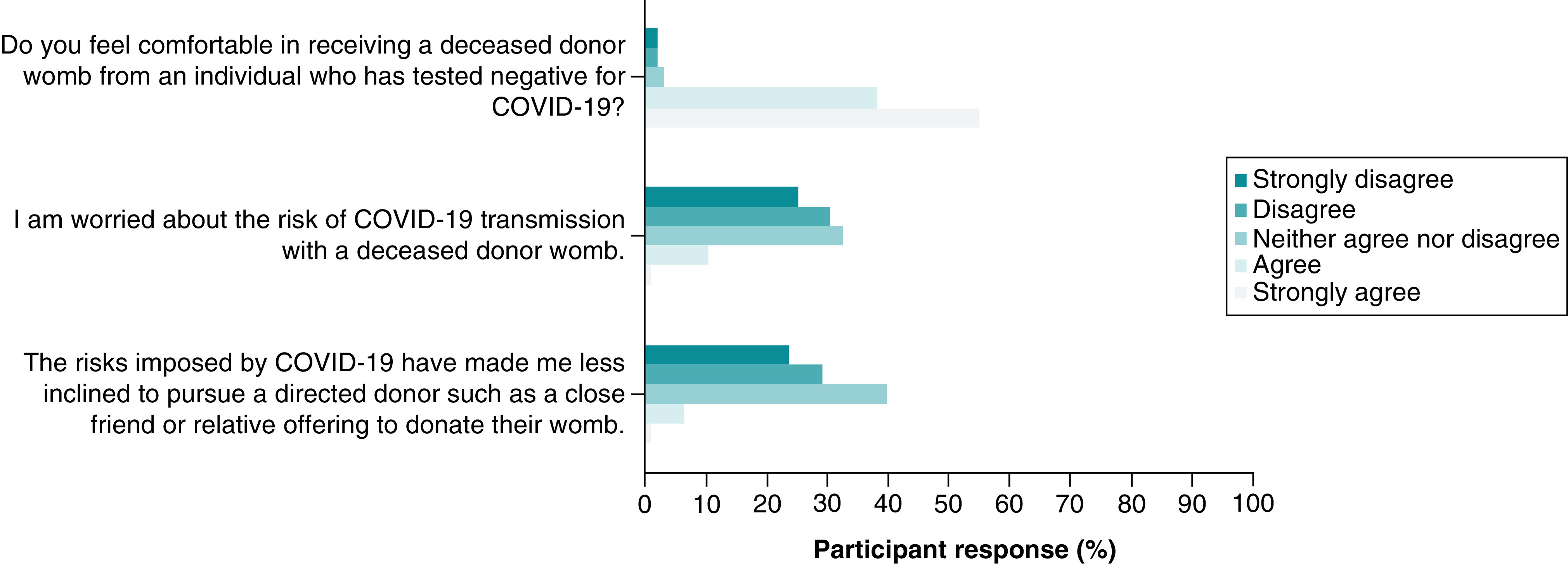
Perceived risk of COVID-19 transmission from uterus donor.

The preferred uterus donor type remained similar to pre-pandemic levels with 60% (n = 56) of women choosing living donor and 40% (n = 37) choosing deceased donor compared with 62% (n = 58) and 38% (n = 35) respectively, following the pandemic. Over half the women (n = 59, 53%) strongly disagreed or disagreed that the risks imposed by COVID-19 made them less inclined to pursue a directed donor such as a close friend or relative offering to donate their uterus (Figure 7). The majority (n = 53, 56%) of women, strongly disagreed or disagreed on being worried about the risk of COVID-19 transmission with a deceased donor uterus (Figure 7). 92% (n = 87) of women strongly agreed or agreed on feeling comfortable receiving a donor uterus from a deceased donor who tested negative for COVID-19 (Figure 7).

**Figure 8.**
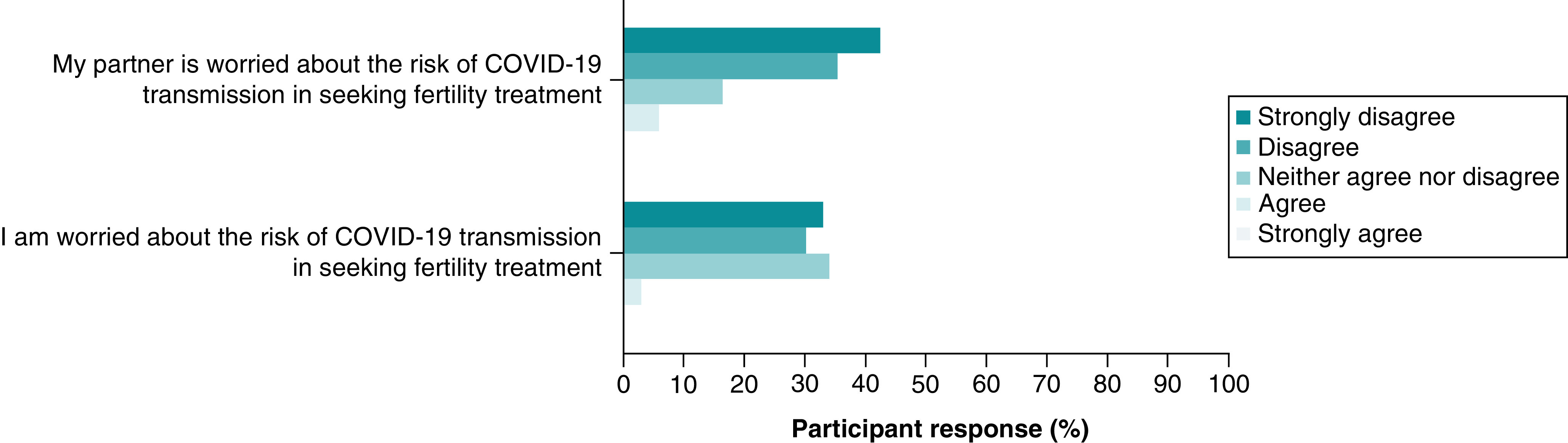
Perceived risk of COVID-19 transmission during fertility treatment.

The vast majority of respondents (n = 57, 63%) strongly disagreed or disagreed that they were concerned about the risk of COVID-19 transmission while seeking fertility treatment (Figure 8). There were 6% (n = 5) of partners who agreed they were concerned on the risk of COVID-19 transmission in fertility treatments (Figure 8). One third of women (n = 29, 33%) strongly agreed or agreed the pandemic had resulted in delays in their fertility treatment. Only 7% (n = 6) of women and similarly 7% (n = 6) of partners strongly agreed or agreed when asked if they put their fertility investigations on hold until the pandemic was over. There were 9% (n = 8) of women who reported the pandemic had resulted in them no longer being eligible for fertility treatment.

## Discussion

### Main findings

Our study revealed a high level of motivation toward achieving parenthood among women with AUFI despite the COVID-19 pandemic. 86% of women were actively pursuing a UTx and 80% of women agreed the pandemic had no impact on their motivation for a UTx. Only 5% of women wanted to delay seeking a UTx until the pandemic is over. The pandemic has had little impact on the preference for the type of uterus donor with 62% preferring a living donor and 38% preferring a deceased donor. The majority of the cohort were not worried about the risk of COVID-19 transmission with a deceased donor uterus (56%), and were comfortable receiving a donor uterus from a deceased donor who tested negative for COVID-19 (92%). Of the women pursuing surrogacy, the majority (85%) had not changed their plans due to the risk of COVID-19 in the surrogate mother. Only 6% of women were more likely to choose adoption due to their concerns on the risk of COVID-19 transmission with surrogacy or a UTx.

### Strengths & limitations

This is the first study to investigate the impact of the pandemic on the desire and motivation for a UTx, thus providing a unique insight onto the strength of the enthusiasm for parenthood in this cohort of women.

A response rate of 56% was achieved which is considered a successful outcome [14]. Considering the cohort, who were all seeking a UTx and most of whom had in previous years received news of not being accepted onto the UK INSITU study, this number is an accomplishment. Additionally, it highlights a degree of ongoing engagement with the team and suggests continuing interest in a UTx as highlighted in the high number of women who specified they were still actively pursuing a UTx.

The demographic in this study was reflective of the UTx cohort to date. In our study, the majority of participants (67%) had MRKH, a similar finding to the international literature which reports 89% of women undergoing UTx hold a diagnosis of MRKH [5]. The age range in majority of the respondents was reflective of the range in recipients of a UTx [5]. The modal age ranges among respondents of 30–39 years is also reflective of required age range for inclusion in the UK INSITU trial thus adding to the validity of the sample being similar to that of a potential UTx recipient cohort. The results of the study align with previous studies prior to the Covid-pandemic, across similar cohorts which highlight a high degree of motivation and willingness to undergo significant risk in achieving a UTx. In a cohort of 281 women with MRKH, 21% of women ranked the personal risk imposed by a UTx as least important when weighing up the benefits and risks of the procedure [15]. In another cohort, 61% of women with AUFI reported they would would still consider UTx to achieve motherhood despite being informed of the potential risks involved [16].

However, the study does have its limitations. Firstly, the cohort consisted of women who had previously contacted the team expressing an interest in a UTx. Potentially, there may have been a bias toward answering questions in the positive to improve their position toward achieving a UTx. Additionally, perhaps the judgement of risk was clouded by the desire for a child, which has been proven in this cohort to be strong. Secondly, the study was conducted after the widespread introduction of the COVID-19 vaccination programme. Health behaviours and perceived susceptibility to COVID-19 have been found to markedly reduce post vaccination which may account for the high levels of motivation to undergo a transplant during the pandemic [17].

### Interpretation

Our assumption of the COVID-19 pandemic having a negative impact on desire for a UTx was disproven. A high number (80%) of the respondents reported no impact which suggests a robust desire for a UTx despite the risks imposed by the environment.

The demographics of our cohort warrant close consideration when evaluating the high levels of motivation for a UTx. There are several factors which may contribute to the emotional distress around infertility, including lower educational level and low socioeconomic status. Although the latter was not assessed in this study, 51% of the cohort reported an educational attainment of level 3 or below. Furthermore, the motivation and desire for children is known to differ between women with different levels of education. Studies have revealed women educated to a primary level are more motivated toward fertility and attaining parenthood compared with women educated to a secondary level [18].

One explanation for the strong desire for a UTx is the high number of women (67%) who identified as being part of a religious group in addition to 43% reporting they were married. Studies report on the high degree of stigma relating to social status and marital wellbeing experienced by infertile women in certain religious groups [19]. Across many religions, parenthood is highly desirable and seen as a natural consequence of marriage [20].

The majority of women (64%) reported they were not concerned about the impact of COVID-19 on the baby born following a UTx. This, perhaps suggests a good understanding among the cohort with respect to the fetal risks and may be suggestive of keeping up to date with published material.

Interestingly, the pandemic has had little change on the preference for the type of UTx donor. Also, the majority of women (92%) were comfortable receiving a donor uterus from a deceased donor who tested negative for COVID-19. These results suggest the cohort is trusting of the pre donation COVID-19 testing process, and comfortable to proceed as they planned to do so pre pandemic.

There was a high level of acceptance of the COVID-19 vaccine among our cohort which was much higher than the national figures [21]. When asked if they would feel more comfortable pursuing a UTx if offered the vaccine, 65% disagreed or remained un-decided. The high level of acceptability may be reflective of the motivation to engage in the required health behaviours in order to achieve the outcome of a UTx.

Given an essential prerequisite to a UTx is IVF treatment and the creation of high-grade embryos, the study explored the impact on fertility treatment. Only 7% of women put their fertility investigations on hold due to the pandemic. Again, highlighting the strong desire for parenthood. The delays caused by the pandemic did result in 9% of women no longer being eligible for fertility treatment, which emphasises the great impact the pause in fertility treatments had on women with a short time-span to qualify for treatment and subsequently a UTx.

Interestingly, the study revealed partners also felt strongly about the continuation of the pursuit of a UTx and fertility treatment despite the pandemic, suggesting a degree of interdependence among the couples. Studies suggest women are able to better cope with the stress and anxiety related to infertility when supported by a partner [22,23]. The majority of women (94%) in our study identified they were in a relationship. The high level of motivation to undergo an invasive procedure such as a UTx during a pandemic may also be attributed to the balance of support provided by the presence of a partner.

## Conclusion

A diagnosis of absolute infertility can be a tremendous burden for women. UTx restores and brings the hope of biological parenthood. The COVID-19 pandemic resulted in a delay in UTx activity in the UK. This study has revealed the pandemic had little impact on the desire for a child and women continue to express a high level of motivation and desire for a UTx despite the risks imposed by the COVID-19 pandemic. Women were not worried about receiving a uterus from a deceased donor and there has been little change post pandemic in the preference toward the type of uterus donor. Partners were also supportive in the continuation of the pursuit of a UTx.

**Summary points**

- The main cohort seeking a UTx are women with absolute uterus factor infertility (AUFI). This group includes women with a congenital absence of the uterus termed Mayer–Rokitansky–Küster–Hauser Syndome (MRKH), women who have undergone a hysterectomy and women with severe Ashermans syndrome. The majority of UTx recipients to date have been women with MRKH.
- The alternative options for women with AUFI to acquire motherhood includes surrogacy and adoption. The emergence of UTx has enabled them to embrace the hope of biological, gestational and legal motherhood. A global pandemic resulting in the halting of non-essential surgery is therefore likely to have and had negative consequences on the hope carried by this cohort.
- Being a quality of life improving transplant procedure, and owing to the uncertainties surrounding risk, the pause on UTx activity during the COVID-19 pandemic was appropriate. However, the often over looked potential psychological implications on such women also required consideration. The once given ‘hope’ of parenthood for women given a diagnosis of AUFI was, during the pandemic, repressed.
- With regards to the impact on their motivation for a UTx, the majority of women felt the pandemic had no impact. Similarly, the majority of women felt their desire for a baby strongly outweighs the risks of undergoing a UTx during a pandemic.
- Most of the women did not wish to delay seeking a UTx until the pandemic is over, with only a minority stating they strongly agreed or agreed to a delay.
- The preferred uterus donor type remained similar to pre-pandemic levels. Most women responded that the risks imposed by COVID-19 did not make them less inclined to pursue a directed uterus donor such as a close friend or relative The majority of women were not worried about the risk of COVID-19 transmission with a deceased donor uterus.
- Our assumption of the COVID-19 pandemic having a negative impact on desire for a UTx was disproven. A high number of women reported no impact which suggests a robust desire for a UTx despite the risks imposed by the environment.
