# Supplementary material for: The impact of COVID-19 on the motivations of women seeking a uterus transplant
Source: Future Sci OA. 2023 Mar 28;9(2):FSO846. doi: 10.2144/fsoa-2022-0047 (PMC10061245; doi:10.2144/fsoa-2022-0047)
Supplement: Supplementary file 1 [file fsoa-09-846-s1.pdf]

Imperial College London

Research Study: Participant Information Sheet

The impact of Covid-19 on of women seeking a womb transplant

We invite you to take part in our research.

This Participant Information Sheet tells you about the research study. It explains the purpose of the study and what is involved. This will help you decide if you want to take part. Please read this information carefully. You can contact the team with any questions if you don't understand or want to know more about the study. Before deciding whether or not to take part, you may wish to discuss this information with somebody else

Key things to know

- This survey is open to all women seeking a womb transplant aged 16+ years.
- The study involves completing an anonymous survey lasting 5-10 minutes.
- Your involvement will not impact your eligibility for a womb transplant.
- If you decide you want to take part, you will be asked to electronically sign a consent form on the online survey.
- All responses will be stored securely, confidentially and treated anonymously.
- The research team and others will not know which responses are yours

Contents

1. What's the purpose of the study?
2. Why have I been invited?
3. Do I have to take part?
4. What will happen to me if I take part?
5. What are the possible benefits to me if I take part?
6. What are the possible disadvantages and risks of taking part?
7. What if something goes wrong?
8. Will taking part be kept confidential?
9. What will happen to the results of the research study?
10. Who is organising and funding the study?
11. Who has reviewed the research study?
12. Further contacts

How to get in touch

If anything is not clear or you have any questions or complaints about the study, please contact the study coordinator: Dr Saaliha Vali. Email: s.vali@nhs.net.

Thank you for reading this leaflet and considering whether to take part in the study

1. What is the purpose of this study?

This is an online survey is part of a research study investigating if the Covid-19 pandemic has impacted the motivations of women seeking a womb transplant and how they feel about the other options for parenthood.

We know individuals in receipt of any organ transplant are more vulnerable towards contracting COVID-19. This is mostly due to the need for immunosuppression medication, the greater exposure to hotspots of infection such as healthcare environments and workers, and the health status of individuals who need a transplant, given they often suffer with a number of medical problems. However, in the context of womb transplantation, women who are young, fit and healthy with no significant medical problems are selected which puts them in a more favourable position with regards to the risk of contracting and recovery from Covid-19. Additionally, being on immunosuppression does place these women in a high-risk category but research has shown individuals on immunosuppression medication can still generate sufficient immunity against COVID-19 and with good outcomes. In general, studies in patients with various organ transplants such as the kidney and the liver have shown good outcomes following Covid-19 infection, with most patients recovering successfully without the need for ventilation. We would like to investigate how women who are potential recipients of a womb transplant feel about undergoing the procedure during this period.

This study includes a questionnaire which aims to assess your background, details of your previous medical history and fertility treatment, your views on the risks imposed upon you undergoing a womb transplant after the pandemic and the views of your partner.

Your responses will help to identify if women seeking a womb transplant have changed their views on it following the Covid-19 outbreak and if there are any concerns or misconceptions the womb transplant team need to address.

2. Why have I been **invited**?

You have been invited to participate because you are a woman who has approached Womb Transplant UK to enquire about womb transplantation or you have previously applied to the womb transplant trial (INSITU trial).

3. Do I have to take part?

It is up to you to decide whether or not to take part. If you do decide to take part, you will be given this information sheet to keep and be asked to sign a consent form. If you decide to take part, you are still free to withdraw before submitting the survey. Once you have submitted the survey all your data is anonymised so there is no risk of you being identified.

4. **What will happen to me if I take part**?

The only thing required is for you is to first read the participant information sheet, following this you will complete the electronic consent form and questionnaire on the surveymonkey.com platform using the link provided. It should take approximately 10 minutes to complete. Following the completion of the questionnaire, no further action will be required. All your data is anonymised so you won't be identifiable. Data will be stored on a password protected computer and a paper copy of the consent form you sign will be kept in a secure office within an NHS facility as per research protocol.

5. What are the possible benefits to me if I take part?

This study will give you the opportunity to express your views towards womb transplantation and in particular your feelings towards receiving a womb transplant following the Covid-19 outbreak. Whilst the results may not directly influence you, your responses, along with the other responses received, will determine the need for our research team to address any concerns or misconceptions.

6. What are the possible disadvantages and risks of taking part?

There are no disadvantages or risks to your partaking in this questionnaire, other than the time taken to complete it.

7. What if something goes wrong?

If you are harmed by taking part in this research project, there are no special compensation arrangements. If you are harmed due to someone's negligence, then you may have grounds for a legal action. Regardless of this, if you wish to complain, or have any concerns about any aspect of the way you have been treated during the course of this study then you should immediately inform the Principle Investigator Mr Richard Smith (james.smith25@nhs.net) If you are still not satisfied with the response, you may contact the Imperial College Research Governance and Integrity Team.

8. Will taking part be kept confidential?

This information will not identify you and will not be combined with other information in a way that could identify you. The information will only be used for the purpose of research, and cannot be used to contact you or to affect you in the future. It will not be used to make decisions about future services available to you, such as insurance.

Imperial College London is the sponsor for this study based in the United Kingdom. We will be using information from you in order to undertake this study and will act as the data controller

for this study. This means that we are responsible for looking after your information and using it properly.

Information on Imperial College London's retention periods may be found at https://www.imperial.ac.uk/media/imperial-college/administration-and-support-services/records-and-archives/public/RetentionSchedule.pdf.

9. What will happen to the results of the research study?

The results from this study will be used directly by the womb transplant research team to investigate the impact of the Covid-19 outbreak on women seeking a womb transplant. The results may be shared in a variety of ways, including shared with the public, published in medical journals and presented at national and international conferences. All data will be anonymised so you will not be identified.

10. Who is organising and funding the study?

This study has been organised by the registered charity Womb Transplant UK (Charity no 1138559). No healthcare professional will receive payment for conducting this research.

11. Who has reviewed the study?

This study was given ethical approval by Professor Vassilios Papalois, Head of Department and the Research Governance Integrity Team (RGIT).

12. Further contacts

Study coordinator: Dr Saaliha Vali Email: s.vali@nhs.net

Principal Investigator: Mr J Richard Smith Email james.smith25@nhs.net
