# Supplementary material for: The impact of COVID-19 on the motivations of women seeking a uterus transplant
Source: Future Sci OA. 2023 Mar 28;9(2):FSO846. doi: 10.2144/fsoa-2022-0047 (PMC10061245; doi:10.2144/fsoa-2022-0047)
Supplement: Supplementary file 2 [file fsoa-09-846-s2.pdf]

<u>The Impact of Covid-19 on Women Seeking a Womb Transplant</u>

Questionnaire (V2 – 05/03/21)

Full title: The impact of Covid-19 on the motivations of women seeking a womb transplant.

Please complete the following questionnaire after reading the participant information sheet

Consent

1. I confirm that I have read and understand the participant information leaflet (V2 05-03-21) have had the opportunity to consider the information. ☐

2. I understand that my participation is voluntary and that I am free to withdraw before submitting the questionnaire without giving any reason, without my legal rights being affected. ☐

3. I understand that the information collected may be used to support other research in the future, and may be shared anonymously with other researchers. ☐

4. I confirm I am aged over 16 years ☐

5. I agree to take part in the above study. ☐

© Imperial College of Science, Technology and Medicine

Section 1: Demographic details

Demographic information

1) **Age (years)** …………………

2) **Ethnicity**
   - White ☐
   - Asian ☐
   - Black ☐
   - Mixed ☐
   - Other ☐
   - Would rather not say ☐

3) **Educational attainment:** please select the highest level of qualification you hold:

   No formal qualification ☐

   Level 1 (1-4 GCSEs, Scottish Standard Grade or equivalent qualifications). ☐

   Level 2 (5 + GCSEs, Scottish Higher, Scottish Advanced Higher or equivalent qualifications). ☐

   Apprenticeship (Apprenticeships (England, Wales and Northern Ireland only). ☐

   Level 3 (2 + A-levels, , HNC, HND, SVQ level 4 or equivalent qualifications). ☐

   Level 4 or above (First or higher degree, professional qualifications or other equivalent higher education qualifications). ☐

   Other qualifications (Other vocational / work related qualifications and non-UK / foreign qualifications (England, Wales and Northern Ireland only). ☐

4) **Employment status**
   - Employed (Full time) ☐
   - Employed (Part time) ☐
   - Self Employed ☐
   - Student ☐
   - Housewife ☐
   - Unemployed ☐
   - Would rather not say ☐

© Imperial College of Science, Technology and Medicine

5) Religion

- Christian ☐
- Muslim ☐
- Hindu ☐
- Other ☐
- Atheist ☐
- Would rather not say ☐

6) Relationship status

- Single ☐
- Living with partner ☐
- Married ☐
- Divorced ☐
- Separated ☐
- Widowed ☐
- Would rather not say ☐

7) What is the cause of your womb related infertility

I have been diagnosed with MRKH ☐
Hysterectomy for the treatment of cancer ☐
Hysterectomy for the treatment of gestational trophoblastic disease ☐
Hysterectomy following heavy bleeding at caesarean section ☐
Hysterectomy for the treatment of fibroids ☐
Severely scarred womb (Ashermans syndrome) ☐

8) Do you have children?

Yes ☐ How many? …………………….
Adaption Yes☐ No☐
Surrogate Yes☐ No☐
Biological Yes☐ No☐

No ☐

9) Medical history

Do you have any medical problems we should be aware of? Yes ☐ No ☐

If yes, please detail below:

Have you previously had cancer? Yes ☐ No ☐

If yes, please detail below:

© Imperial College of Science, Technology and Medicine

Do you have any mental health issues we should be aware of?
Yes ☐ No ☐

If yes, please detail below:

10) Relationship status

Single ☐

In a relationship <1yr☐ 1-2years☐ 3-4 years☐ 5+years☐

Section 2: The impact of Covid-19 on your desire for a womb transplant.

11) Which of the following options are you planning or actively pursuing? *(tick all that apply*)

Womb transplant ☐

Surrogacy ☐

Adoption ☐

None ☐

12) Are you still considering a womb transplant?

Yes ☐

No ☐

Undecided ☐

13) What impact has the pandemic had on your desire for a pregnancy?

More motivated ☐

Less motivated ☐

No change ☐

With regards to surrogacy:

14) I feel anxious about the surrogate mother exercising enough caution during her pregnancy

Strongly agree ☐ Agree ☐ Undecided ☐ Disagree ☐ Strongly Disagree ☐

15) I have changed my plans for pursuing surrogacy due to the risk of Covid-19 in the surrogate mother.

Strongly agree ☐ Agree ☐ Undecided ☐ Disagree ☐ Strongly Disagree ☐

With regards to adoption:

16) I am more likely to pursue adoption following the pandemic due to concerns about the risk of Covid-19 transmission with surrogacy and womb transplantation.

Strongly agree ☐ Agree ☐ Undecided ☐ Disagree ☐ Strongly Disagree ☐

17) I am less likely to pursue adoption following the pandemic due to concerns about the risk of Covid-19 transmission.

Strongly agree ☐ Agree ☐ Undecided ☐ Disagree ☐ Strongly Disagree ☐

Regarding the Covid-19 pandemic:

18) I am now less motivated in seeking a womb transplant due to the increased risk posed by Covid-19.

Strongly agree ☐ Agree ☐ Undecided ☐ Disagree ☐ Strongly Disagree ☐

19) There has been no impact on my motivation for a womb transplant

Strongly agree ☐ Agree ☐ Undecided ☐ Disagree ☐ Strongly Disagree ☐

20) I would like to delay seeking a womb transplant until we are clear of the pandemic

Strongly agree ☐ Agree ☐ Undecided ☐ Disagree ☐ Strongly Disagree ☐

21) I would like to delay seeking a womb transplant due to concerns regarding the transmission of Covid-19 during pregnancy

Strongly agree ☐ Agree ☐ Undecided ☐ Disagree ☐ Strongly Disagree ☐

© Imperial College of Science, Technology and Medicine

22) My desire for a baby far outweighs the risks of undergoing a womb transplant during the pandemic

Strongly agree ☐ Agree ☐ Undecided ☐ Disagree ☐ Strongly Disagree ☐

23) I am worried about the impact of Covid-19 on the baby born following womb transplantation

Strongly agree ☐ Agree ☐ Undecided ☐ Disagree ☐ Strongly Disagree ☐

24) I would feel more comfortable pursuing a womb transplant if offered the vaccine

Strongly agree ☐ Agree ☐ Undecided ☐ Disagree ☐ Strongly Disagree ☐

25) I would accept to have the Covid-19 vaccine prior to undergoing womb transplantation

Strongly agree ☐ Agree ☐ Undecided ☐ Disagree ☐ Strongly Disagree ☐

Section 3: The impact of Covid-19 on the choice of womb donor

26) In seeking a womb transplant which is now your preferred option?

Living donor ☐

Deceased donor ☐

27) What was your preferred option prior to the Covid-19 pandemic?

Living donor ☐

Deceased donor ☐

28) The risks imposed by Covid-19 have made me less inclined to pursue a directed donor such as a close friend or relative offering to donate their womb.

Strongly agree ☐ Agree ☐ Undecided ☐ Disagree ☐ Strongly Disagree ☐

29) I am worried about the risk of Covid-19 transmission with a deceased donor womb.

© Imperial College of Science, Technology and Medicine

Strongly agree ☐ Agree ☐ Undecided ☐ Disagree ☐ Strongly Disagree ☐

30) **Every potential deceased organ donor is being tested for COVID-19 and if they test positive they will not be able to donate. Do you feel comfortable in receiving a deceased donor womb from an individual who has tested negative?**

Strongly agree ☐ Agree ☐ Undecided ☐ Disagree ☐ Strongly Disagree ☐

Section 4: The impact of Covid-19 on your fertility treatment

If you have applied for fertility treatment:

31) **How long have you been waiting for an appointment?**

≤ 2 weeks ☐
3-4 weeks ☐
5-6 weeks ☐
7-8 weeks ☐
9-10 weeks ☐
11-12 weeks ☐
12+ weeks ☐
Not applicable ☐

32) **I am worried about the risk of Covid-19 transmission in seeking fertility treatment**

Strongly agree ☐ Agree ☐ Undecided ☐ Disagree ☐ Strongly Disagree ☐

33) **The pandemic has resulted in delays to my fertility treatment**

Strongly agree ☐ Agree ☐ Undecided ☐ Disagree ☐ Strongly Disagree ☐

34) **The delays caused by the pandemic have resulted in me no longer being eligible for fertility treatment**

Yes ☐ No ☐

35) **I have managed to have my eggs/embryos frozen during the pandemic**

Yes ☐ No ☐

If yes please say which: egg/embryos …………………………..

36) **I have personally decided to put all investigations on hold until the pandemic is over**

© Imperial College of Science, Technology and Medicine

Strongly agree ☐ Agree ☐ Undecided ☐ Disagree ☐ Strongly Disagree ☐

37) I am worried about the delay in fertility treatment resulting in an unsuccessful outcome

Strongly agree ☐ Agree ☐ Undecided ☐ Disagree ☐ Strongly Disagree ☐

Section 5: The impact of Covid-19 on your partners views

38) I have a partner ☐
I do not have a partner ☐ *(Please skip to Section 6)*

39) My partner now feels we should not pursue a womb transplant due to the risk imposed by Covid-19.

Strongly agree ☐ Agree ☐ Undecided ☐ Disagree ☐ Strongly Disagree ☐

40) My partner would like to delay seeking a womb transplant until we are clear of the pandemic

Strongly agree ☐ Agree ☐ Undecided ☐ Disagree ☐ Strongly Disagree ☐

41) My partner is worried about the risk of Covid-19 transmission in seeking fertility treatment

Strongly agree ☐ Agree ☐ Undecided ☐ Disagree ☐ Strongly Disagree ☐

42) My partner feels we should put all investigations on hold until the pandemic is over

Strongly agree ☐ Agree ☐ Undecided ☐ Disagree ☐ Strongly Disagree ☐

Section 6: The impact of Covid-19 on you

43) I have previously had a positive Covid-19 test

Yes ☐ No ☐

© Imperial College of Science, Technology and Medicine

44) If you have previously been affected by Covid-19 do you feel you suffering with any long-term effects?

Yes ☐ Please state……………….

No ☐

Not applicable ☐

45) Outside of the womb transplant programme, if offered the Covid-19 vaccine would you accept to take it?

Yes ☐ No ☐ Don't know ☐

~The end~

© Imperial College of Science, Technology and Medicine
